# The Burnout Phenomenon: A Résumé After More Than 15,000 Scientific Publications

**DOI:** 10.3389/fpsyt.2020.519237

**Published:** 2020-12-09

**Authors:** Andreas Hillert, Arnd Albrecht, Ulrich Voderholzer

**Affiliations:** ^1^Schön Klinik Roseneck, Prien am Chiemsee, Germany; ^2^Munich Business School, München/Munich Business Coaching Institute, München, Germany

**Keywords:** burnout, depression, concepts of mental illness, subjective disease models, work-related disorders, scientific conceptualization of psychic phenomena, stress

## Abstract

The “burnout” phenomenon, supposedly caused by work related stress, is a challenge for academic psychiatry both conceptually and professionally. Since the first description of burnout in 1974 until today, more than 140 definitions have been suggested. Burnout–symptomatology's main characteristic, the experience of exhaustion, is unspecific. Different development–models of burnout were proposed, assumed to depict a quasi-natural process. These could not be confirmed empirically. An expert consensus on the diagnostic criteria and the conceptual location, whether as an independent disorder or as a risk, could not be agreed on. Nevertheless, the phenomenon of burnout in the ICD-11 is considered to be categorized as a work-related disorder. Psychiatric research on the burnout–phenomenon ignores problems of definition resulting from different perspectives: It may meet societal expectations, but does not fulfill scientific criteria, and therefore is not suitable to establish an objective diagnosis and treatment. Parallel detection of ICD/DSM diagnoses from an expert perspective and subjective perturbation models are considered appropriate.

## Introduction

Since the first publication on the burnout topic 1974, in which the psychotherapist Herbert Freudenberger literally described the phenomenon in relation to his own body and mental state, an infinite amount on the subject of burnout has been published (1). Burnout was first recognized in social professions, later in all occupational related subjects where people experienced stress and in situations of perceived overwork. In these contexts “burnout” was discussed and expected to be treated, preferably by methods aiming for relief, rest and relaxation (2, 3). There are more than 140 suggested burnout definitions in the literature (2, 4). Nevertheless, the hope that “burnout research” in psychiatry, psychology, psychotherapy, neurophysiology and the social sciences will find a concrete concept that is viable for various scientific and therapeutic issues and ultimately also for political implications has not been fulfilled. Concrete diagnostic criteria to categorize burnout as a disorder according to DSM or ICD standards does not exist to this day. Often references are made to burnout questionnaires, in particular to the Maslach Burnout Inventory (MBI) (5). Although a lot of authors struggle with the burnout definition, many of them believe to know what burnout is and publish articles about it (“I can't define it, but I know what it is!”). This leads to the questions on which this article is based: How realistic is it to come closer to an unambiguous medical-psychotherapeutic definition or even standard? Is a clear definition of burnout even possible–based on established scientific criteria? To what extent can consideration of the respective research or observer perspective, i.e., the perspective from which burnout is perceived or processed, contribute to clarifying the question?

A systematic review on the entire burnout research history since 1974 and more than 15,000 references on the subject (in PubMed 17,836 citations are available, October, 28nd 2020) is not possible to streamline in this paper.

In order to at least indicate the heterogeneity and distribution of the burnout-related questions characteristic of the topic, here is a list of the contents and questions of the last publications on the topic listed in medline. On medline, February 10.02.20, the 100 most recent publications on burnout (in the sense of a psychic phenomenon) were as follows: 57 studies based on surveys of circumscribed groups of doctors, students, teachers, administrative staff, etc., partly with the question how burnout values are related to other recorded parameters; 31 discussion contributions on the subject without own data; 7 prevention or therapy studies (mostly based on mindfulness based stress reduction); 3 reviews on burnout-exposure and changes in special groups, and 3 methodical works (evaluation of burnout questionnaires). Most of the empirical studies are using the Maslach burnout inventory or related instruments. The data are used to show and discuss the particular relevance of special group's burdens and to consider possible solutions.

In this paper an exemplary selection of publications depicting the spectrum of publications on the topic is quoted with the aim of showing the urgency of standardizing the definition of burnout for scientific and therapeutic procedure.

## Material: Scientific-Conceptual, Therapeutic and Personal Perspectives

Freudenberger introduced the “burnout”–phenomenon in 1974 (1). Since then, numerous more or less scientifically designed, more or less professionally accentuated studies on the subject have been conducted and published. Here already the problem starts with the fact that authors of scientific papers about burnout do not usually define burnout or at least not explain in which category the burnout definition is allocated.

**Figure 1:**
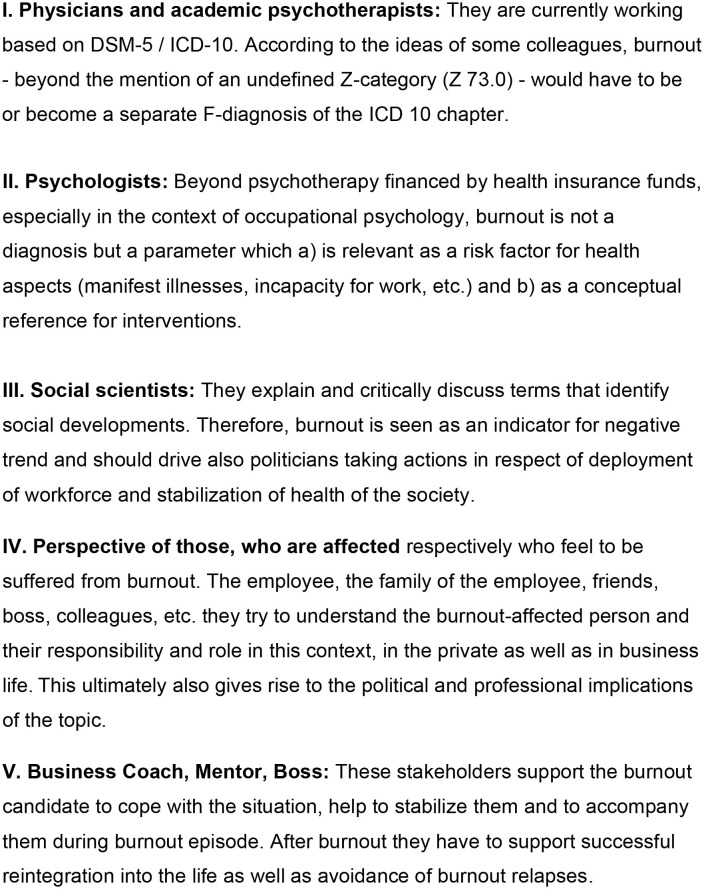
Perspectives on burnout.

The definition and description depend on the authors' business perspective, profession and his personal point of view as pointed out elsewhere (6). They are correspondingly highly heterogeneous (Figure 1).

### Medical and Psychotherapeutic Perspective

Diagnoses should be objective, reliable and valid. Ideally, different examiners will recognize the same central symptoms (objectivity) in a patient. Findings must be reproducible and reliable, i.e., confirmed by retests. A diagnosis is valid if it depicts a disease that can be treated with a certain method and/or can be traced back to a defined etiology. This is not usually the case with mental disorders (biopsychosocial phenomena). Robert L. Spitzer (1932–2015) was clear about this when preparing the DSM-III (7, 8). As long as one started from etiological models (for example, “neurotic” vs. “endogenous” depression), the diagnosis could be made by reading tea leaves. Since the DSM-III, psychiatric disorders have been defined based on symptoms and of course, the reliability has increased and is regarded as a milestone in psychiatric research. The ICD diagnostic system has followed this logic. Based on this, what would a burnout diagnosis look like? Table 1 calls the most frequent burnout symptoms according to literature:

**Table 1:** Burnout symptoms [based on Burisch (4, 9)].

• Exhaustion, lack of energy, sleep disorders • Concentration and memory problems, feelings of insufficiency, inability to make decisions • Reduced initiative and imagination, indifference, boredom, disillusionment, inclination to cry, weakness, restlessness, despair • Greater distance from clients, emphasis on jargon, accusations against others, loss of empathy, cynicism, loss of idealism, bitterness, “dehumanization” • Partnership and/or family issues • Feeling of lack of recognition • Physical symptoms such as: tightness in the chest, difficulty breathing, back pain, nausea, increased nicotine and alcohol consumption

The reason why burnout cannot be diagnosed on the basis of symptoms is:

1. All symptoms according to Burish–and many other authors–are non-specific. For example, symptoms like exhaustion, concentration problems, and reduced initiative could be caused by a viral infection, hypothyroidism, or low blood pressure.
2. No specific symptom combination or syndromes can be identified. According to this Herbert Freudenberger, in the very first essay on the subject (1), assumed that the symptoms of burnout were different for each person affected and proposed defining burnout based on etiology: He supposed burnout to be the result of occupational overload in previously committed individuals. Better working conditions and coaching might be an effective and efficient response (10). The perception that burnout is the result of long-term occupational overload appears particularly plausible and imperative if you assume that people work like batteries: the more demand is placed on them, the faster their energy is drained. On the other hand, such causal connections are methodologically difficult to detect and do not solve the definition problem.
  - Is anyone who feels overloaded a burnout-case?
  - In what context are the demands, the individual coping strategies and the motivation to cope with these demands?
  - To what extent do non-sensical work tasks lead to burnout experience?
  - How is the experience of effort-reward imbalance associated with burnout constellations? (11).

Since 1990 at least, there have been studies that show that it is less the “dedicated” employees who burn out (12). Many years of work in stressful occupations does not necessarily increase the burnout risk (13). Empirically, younger people experience burnout more often than older ones. This is then hardly compatible with the “battery model” of burnout (14, 15).

**Figure 2:**
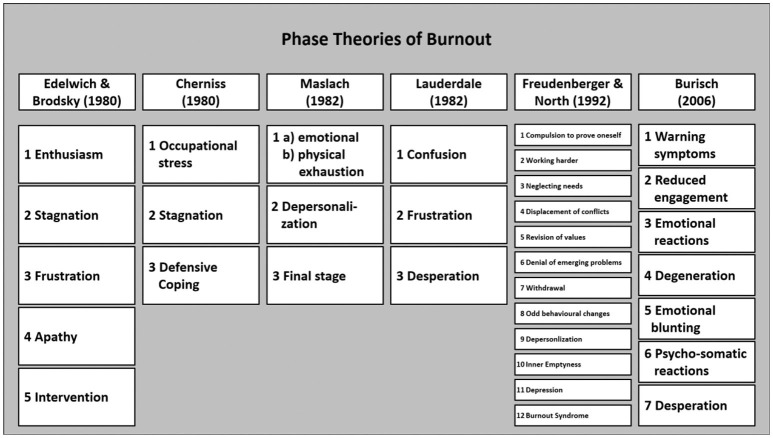
Overview on most common burnout phase theories.

Herbert Freudenberger and many others divide the burnout course into stages or phases. At least the DIMDI Report of 2011 (16) shows how heterogeneous the authors' opinions are regarding the development of burnout. Two to more than twelve stages were proposed (Figure 2).

Here, there is little attempt to operationalize and demarcate the various stages: Who does not occasionally feel exhausted, helpless, unsuccessful and less friendly? In any case, follow-up studies show that regular “burnout processes” do not exist. People have quite stable patterns of deal with occupational demands (17).

### Burnout or Exhaustion or Depression?

The question of what distinguishes the symptoms of burnout and depression, and how the two phenomena can be distinguished from each other, has been discussed extensively (18–21). Attempts to differentiate between burnout and depression only convince on theoretical level (22). Therefore, for instance, an inadequate “adaptation to high stress,” is a burnout risk factor is an insufficient “adaptation to aversive pressures” in depression risk. Lack of mental drive may lead to exhaustion, or vice versa. In a depression, this is defined as “motivation deficits.” Evaluation has never been established by the authors so far. Endogenous (morning depression, feeling of numbness, etc.) and neurotic depression are hardly distinguishable. Therefore, it cannot be used as clear diagnostic criteria. Should an episode of lack of drive experienced as an exhaustion or to be caused by a lack of motivation?

Whether exhaustion is caused by a lack of drive or a lack of motivation, probably reflects the introspection patterns of those affected. In any case, it can be assumed that a reliable differentiation of the constellations outlined here is ultimately impossible!

With the Position-Paper of 2012, the German Association for Psychiatry, Psychotherapy and Psychosomatics (German Association for Psychiatry, Psychotherapy and Psychosomatics–DGPPN) claimed the burnout interpretive sovereignty in German-speaking countries (23). Burnout is defined as a risk state in the transition area of (still) acceptable stress and already manifest of diseases/disorders. Unfortunately, the position paper does not mention any corresponding criteria or symptoms. What is beyond an experienced fatigue in the face of chronic stress, could be characterized by risk factors? And what would be the added value of this, since the phenomenon of chronic stress alone has been shown to be a health risk factor already (24, 25). Should every stress-risk state be named burnout, no matter how healthy or ill the concerning person is?

### Psychological Perspective

Is burnout what is measured by a burnout questionnaire? In this is the case, it is easier for psychologists than medical doctors and psychotherapists to define burnout. According to Christina Maslach, burnout is characterized by emotional exhaustion, depersonalization, and reduced capacity (occupational overload) (5, 26). The more MBI items are approved, the more burned out you are. The objectivity and reliability of the questionnaires had several times be statistically proven by (5).

But until today, there have been no representative MBI-reference values established and the validity has never been proved (27). Various burnout questionnaires have been developed which are similar to the MBI, such as the Tedium Measure/Scale. Most of these did not gain acceptance as research instruments (28). In addition to the MBI, currently the questions in the Copenhagen Burnout Inventory are (often) discussed in the scientific literature (29, 30).

On the one hand, the structure of burnout (as described in the literature) leads to an adequately depict work-related stress. On the other hand, it is assumed that burnout is suitable for predicting health status. Obviously high burnout values correlate in the middle range with depression and anxiety, with professional dissatisfaction, etc. (31, 32). Results of burnout questionnaires compared with those e.g., of depression questionnaires, have often been published. However what lies behind these findings? Just a glance at the items used to measure burnout answers this question: most of the questions in burnout questionnaires capture aspects that are symptoms of mood disorders. In the same question, the participant should then decide whether these symptoms are due to occupational burdens or not. The questions of burnout questionnaires mix symptom assessment and causal attribution. Burnout questionnaires thus do not measure objective work-related burdens and their consequences, but subjective perception of symptoms and subjective causal attributions! From a methodological point of view, it would therefore not be permissible to conclude from high rates of burnout directly to objective overload or health-endangering working conditions.

For example, a quarter of Bavarian students becoming a teacher have “burned out” before they were even confronted with real teaching experience. It would be appropriate to offer these students already stress management training during their study program (27, 33).

The standard in the everyday life of current burnout research is to question circumscribed groups with the MBI or another burnout-instrument. In each case, it is hypothesized that these very groups are particularly burdened, which is then usually confirmed in a differentiated form. Traditionally, social professions, particularly teachers and physicians, continue to be at the center of this internationally driven research interest. Over the last 15 years, many investigations have been published about burnout among teachers (34–40) and physicians (41, 42). Often the researchers themselves are members of the profession which has been investigated: All of the studies on burnout by doctors cited in this article come from employees of medical institutes; half of the cited studies on teacher health are done by employees of educational institutes, the others by employees of institutes in the mental health framework (focusing on a relevant study population that is claimed to be particularly vulnerable to burnout). The fact that the selection of the respective study population is not coincidental and ultimately also involves (professional) political aspects is scientifically difficult and should be taken into account when interpreting them.

For several years, causes of burnout and possible correlation to neurophysiological parameter has been investigated (43, 44). The findings are heterogeneous, and a practice-relevant summary is difficult. The patterns associated with burnout in a group comparison of those less afflicted (parallel control group), point to chronic stress. Some of the results are similar to those obtained in depressed patients, others are not. The samples are usually small and almost always–in terms of professional group, etc.–selective. As long as the burnout criteria are vague, even with subtle neuropsychological methods, no groundbreaking results can be achieved: Depending on the (design of the) study different aspects of chronic stress and depression are measured. “Large prospective cohort studies examining both conditions in parallel rigorously controlling for confounders are required to further elucidate the differences and similarities of the HPA axis in MDD and the burnout syndrome,” as quoted by a leading group of scientists in this field (44), for the reasons mentioned, cannot lead to further results.

### Social Science Perspective

In postmodern society and the world of work, individual social ties and security (45), technical and social (including the half-life of knowledge content and values) are demanding and stressing the individual (46, 47). With regard to property and power structures, Zygmunt Bauman characterized these phenomena with the term “liquid modernity” (48). From this perspective, burnout is impressive as a logical consequence of the performance-based company that has reached its limits. Academic social scientists diverge strongly in terms of their perspective and weighting of individual factors: to bring the effects of postmodernism on the individual to a certain extent reflected in burnout (49). To what extent this promotes a differentiated social scientific discourse, has to be clarified within the researchers' community.

### Burnout-Affected Perspective

Herbert Freudenberger, although he was a psychological psychotherapist himself, wrote his first essay on the subject, published in 1974, from the perspective of those affected. He was able to flee Germany from the Holocaust, went to New York, studied psychology and worked as a psychotherapist. He worked more than 10 h a day to earn enough money for his family. After this, at night, he worked as a volunteer for socially disadvantaged people. In the face of such working hours, he experienced burnout at first hand. He wrote the famous first essay being apparently unaware concerning his very own biographical motives behind his destructive working behavior (a) security at any price for his family and (b) to support young people, because he himself had been in a similar situation after leaving Germany during the war. Freudenberger (1) was convinced that he had no “neurosis.” And he was convinced that people with burnout do not need a therapist but better working conditions. As a psychotherapist he was an expert. But he experienced and published papers on burnout from the perspective of a person concerned, i.e., as a patient! The picture of a burned-out house fit his concept, (exhaustion, physical complaints, etc.). Burned out: that is exactly how he felt. Expert standards, objectivity and reliability were irrelevant to Freudenberger's burnout-feelings. Burnout was and is individually “discovered” and “suffered” by those affected. The expert perspective on burnout is secondary, attempting to meet the feelings of the “affected person” and at the same time to meet the internal standards of his discipline. In practice this cannot work.

### When Expert Perspectives Get Mixed Up

If authors unthinkingly switch from their primary to other burnout perspectives, it becomes methodically critical: therapists become social scientists, boldly criticizing societal aberrations. Psychologists who misunderstand burnout as a diagnosis build on neurophysiological research. Social scientists argue that burnout is a stress-related disease (50). Conversely, it is like in the fairy tale “The Emperor's New Clothes”: no matter which expert's perspective you consider in terms of burnout, as soon as you do so in a naively critical way, it becomes obvious that the emperor is naked. Moreover, the unabated popularity of the burnout paradigm is surprising. It is precisely this constellation that characterizes the burnout research itself, as it were, “burned out”: if the respective perspective of an author on the topic is not explicated, another 15,000 publications will not lead to a sustainable burnout concept. The background to the persistently high popularity of the burnout phenomenon is foreseeable complex. Perhaps most important: a concise, immediately understandable picture is used. Since the symptoms are unspecific, practically everyone can identify with burnout, the socio-political implications are far-reaching, among other things (as discussed in the following section). The dimension, which ultimately affects everyone themselves, and the breadth of the term apparently make a scientifically abstract approach to the topic fundamentally difficult.

## Burnout: A Self-Concept!

**Figure 3:**
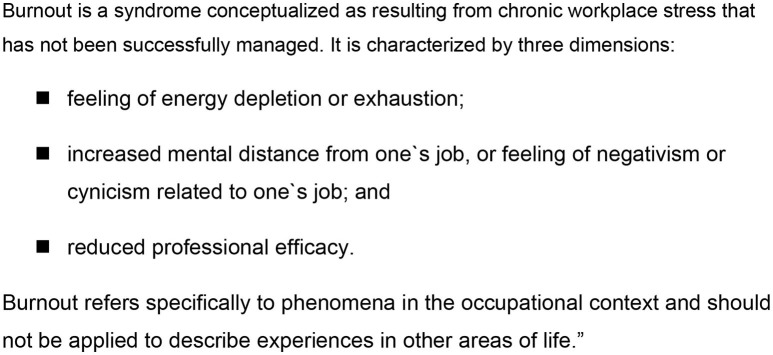
ICD-11 for mortality and morbidity statistics (54).

Why is there no definition of burnout that is capable of consensus in the sense of scientific criteria? Why, despite excessive research in this area (>15,000 publications), has it not been possible to develop a workable concept in this regard? Ultimately, Herbert Freudenberger answered this question in his first essay on the subject, published in 1974*: “Anyone who has ever seen a burned-out house knows how devastating it looks”*. Freudenberger associated a concise picture to characterize his state of health (51). Any attempt to question this image inevitably leads to–scientifically speaking–untenable dimensions. If burnout was taken literally it would be a process that ultimately leads to destruction of nerve cells. Neurophysiological findings indicate that there may be abnormalities (52, 53). However, substantial brain damage looks different. At the same time, burnout postulate that exhaustion manifests itself in work-related contexts rather than in private life (ICD-11—Figure 3). This reflects the area in which burnout has been located in the past decades, based on a “working population” who preferably experiences stress there.

But why and how should professional, not private stress lead to burnout or specific damage (to the brain)? Or is burnout a dissociative disorder? Old fashion established hypothesis that burnout only meets particularly committed persons (55) are disrespectful and not acceptable! As already mentioned, considerations of this kind, which would be important from an expert's perspective, play no role in subjective perturbation models. Speaking at a Burnout Support Group meeting, a spokesperson summed it up: “*I do not care how experts define burnout, I know what it is, I feel it.”*

Finally, the following aspects determine burnout as a self-concept:

- symptoms become causally understandable, referring to models that are established in the respective social group (“*too much work, the batteries are empty”*).
- emotional relief: “the pointer of guilt” does not point to oneself but to the circumstances, work overload, a bad boss, the company, the negative developments in the working world.
- problems can be communicated with poor risk of stigmatization, respectively, with some aspects of self-increasing values.
- and, last but not least, burnout ideas provide a guide to treatment and all that the patient desperately needs: stress reduction, support, recovery, etc. hoping that an early retirement can be averted.

“*There is nothing left. I was much more committed than others. I need to rest. I have to pay attention to myself. I just burned out” - that's why I fell ill with burnout*.

Burnout is a currently convincing subjective disorder model. No more but no less. Burnout is more than a “fashion diagnosis” and points out that the DSM/ICD approach bypasses patient needs. A DSM/ICD-10/11 diagnosis says someone has the symptoms he reported and does not provide any additional information regarding causes, prognosis and therapy. In view of this, it is not surprising that people who experience themselves at the limit of their resilience in the face of professional and other stresses find themselves appropriately in the concise image of “burnout.” And burnout questionnaires? As discussed above, they capture a mixture of symptom experience together with burnout self-identification, such as “*I feel drained from my work”* (MBI). As long as the majority of the population experiences work stress, many symptoms will be explained spontaneously as caused by stress (56). The burnout-Emperor (we have met him already in the fairy tale), does not have any clothes but a highly cotemporary image.

### Burnout Experience and Depression

**Figure 4:**
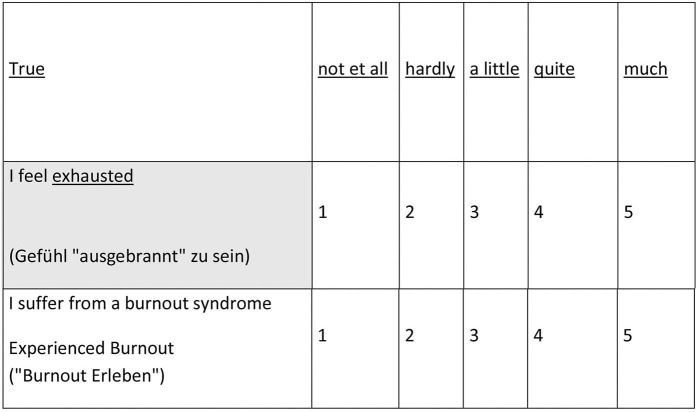
Burnout self-identification questionnaire.

The fact that burnout is genuinely a subjective disorder model does not mean that this cannot be a subject of empirical research. Burnout is a subjective “disturbance model” and can be examined as such. Practically, burnout-self-identification has to be asked for directly. This was realized as part of a large online survey (“Stress Monitor Project”) (57). On behalf of a company health insurance fund, an instrument was developed which was also used in a project with the Bavarian civil service. Currently, more than 40,000 data sets are available. In addition to basic social and occupational data, the stress monitor includes a screening sheet (Depression, anxiety disorders and stress: DASS) (58). In addition, the gratification experience is recorded (59). Self-identification with the terms burnout and “ausgebrannt”—the German terms for burnout—were asked for in the survey conducted in Germany (Figure 4) [for a methodically similar approach to capture burnout see (26), an overlap in terms of memory bias (60)]. After entering the data (processing time 5–9 min.) the participants immediately receive personal feedback. The client receives an anonymized summary at defined times.

**Figure 5:**
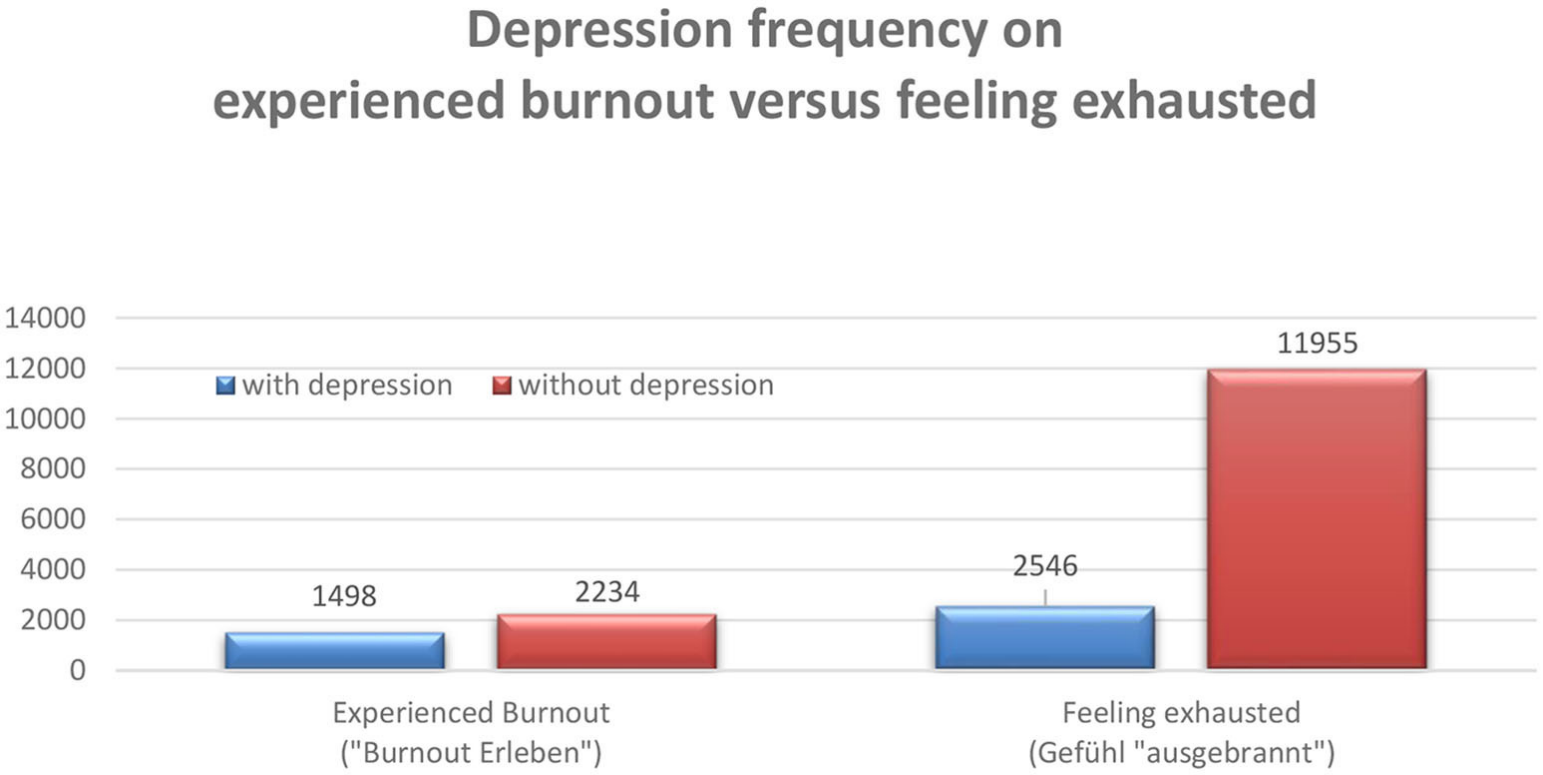
Depression incidences experienced burnouts vs. feeling exhausted (57, 61).

About every second person who feels affected with burnout, but only one in five who feels “ausgebrannt” meets the screening criteria for depression (the results are similar in relation to panic disorder) (Figure 5).

Obviously, from the perspective of German interviewees, the term burnout is more than a translation of “ausgebrannt.” For German people burnout is perceived as a “technical term.” Because of this, people who feel more serious affected, tend to come closer to what the expert understands as “depression.” If the stress monitor data are differentiated according to occupational groups there are clear differences regarding the burnout-identification patterns. This is how, for example, teachers feel more burdened occupationally than employees in a large electrical company, but at the same time experience less burnout. As expected with a subjective model of perturbation, patterns communicated in society, the media, and the respective reference group have an influence on whether and how perturbation models are experienced as individually appropriate and are referred to as discomfort. Overall, with significantly varying, all conceivable constellations can be found: Depressed subjects who experience themselves as “ausgebrannt” and not burned out, non-depressive who experience burnout and not as “ausgebrannt” (rare) etc.

### Summary and Scientific Perspectives

Burnout *per se* only works as subjective model of disruption. Used as medical or psychotherapeutic expert category, burnout remains a blurred, natural-scientifically useless “diagnosis.” Burnout cannot be reliably and validly diagnosed. Notwithstanding the image of burnout is apparently so self-evident that it immunizes some experts against burnout-relativizing arguments. If it were accepted that burnout is a subjective phenomenon and could not been captured in DSM/ICD diagnoses or in a quasi-scientific category, the burnout discussion would have lost some of its attraction but gained some medical-therapeutic basis. The attempt of some experts to refer burnout solely to professional work in the ICD-11 (Figure 3) obviously obscures large parts of the discussion outlined here and will not be successful because burnout is the experience of those affected.

As explained, burnout was and is genuinely a subjective disorder model that, due to its conciseness and public reputation, has been “adopted” in the course of various scientific disciplines, from industrial and organizational psychology to psychiatry. From the point of view of the history of science, it is remarkable that a scientific paradigm, the stress model developed by Hans Selye, was transferred from “those affected” to a problem constellation that is diffuse in terms of symptoms, but frequent in the social context of achievement societies, in order to address them in the given social context to make it understandable and manageable. Seen in this way, burnout is a modern variant of lay models that have by no means lost their function due to the successes of medicine and psychotherapy (62, 63), but rather develop dynamically, reflecting current scientific paradigms, and vice versa have a retroactive effect on–supposedly purely scientific–paradigms. In the context of the discussion about disorder and disease models (64–66), appropriate consideration of this dynamic aspect of the relevant terms would be important; The conceptualization of personalized medicine will foreseeably become even more complex as a result (67).

The burnout burden of students has been intensively researched for several years (37, 68, 69). Surprisingly, high school students measured by the MBI in a student-version (70) experience significantly more burnout than students at University (71, 72). Students who do not know which occupation they want to take later feel considerably (and statistically significantly) more burned out than those who named specific career goals. In such cases, which are becoming increasingly common in postmodern societies, burnout therapy would not reduce the amount those affects allowing them to recover, but rather to enable them to clarify their perspectives (business coaching might have positive effects) (73, 74).

The MBI relationships with depression and workload must be interpreted to be pseudo-correlations already created in the item texts. Based on empirical data, neither the risk status model of DGPPN nor a categorical separation of burnout and depression postulating concepts can be confirmed. There are intersections but also discrepancies between the expert (depression) and the burnout-affected perspectives, whereby the affected person's perspective is highly socio-culturally determined. The discrepancies could only be resolved if either experts unconditionally took over the patient's perspective *(“Everyone who feels burned out is burned out!”*). This would have advantages: patients would feel understood and experience therapists as competent and empathetic. Alternatively, experts could deny the patient's competence to feel burnout, according to the motto: “*I decide based on defined criteria whether you have burnout or not*!,” what is of course non-sense. Both scenarios would be grotesque. Therefore, it is important to understand, why burnout occurs and what the patient's perspective is toward his/her situation and future perspective as well as the environment. It is necessary to support people with burnout by coaching (10, 75) and guiding them on the one hand and with medical/psychological/psychiatric aid on the other hand to establish whether manifest depression or other disease could have been diagnosed.

Burnout is neither an independent diagnosis nor a risk stage. It is a “subjective disorder model” and as such belongs to a fundamentally different category than diagnoses conceptualized from an expert's perspective. Burnout reflects the experience of symptoms of any kind, which are experienced as a result of overload, especially in the professional field. The relevant individual criteria depend, among other aspects, on the social and occupational group-specific framework conditions of an individual. On the other hand, an assessment of whether the same individual is at risk of health or sick is done from an expert perspective. There are overlaps between the experience of the affected and expert assessments. However, there cannot be “transitions” or “intermediate stages” simply because there are different perspectives, which in turn are based on different framework conditions and criteria. Experts' perspectives are hypotheses that can be right or wrong. Perspectives of affected persons are correct *per se*, they correspond to a subjective location without explicit criteria, which can fluctuate depending on the changing context.

The burnout experience, meaning and importance within society underlies a change of the socio-cultural environment. However, from our point of view, it makes no sense to continue researching a conceptualization of burnout as a diagnosis. It is a subjective phenomenon whose value lies in the better acceptance of psychological limitations by those affected. Burn-out can therefore contribute to de-stigmatization and facilitate access to therapy for those affected. In addition, burnout reflects negative developments in the world of work and society. As a socio-scientific and science-historically, exciting phenomenon it should be further explored and discussed as such.

## Data Availability Statement

The original contributions presented in the study are included in the article/supplementary material, further inquiries can be directed to the corresponding author/s.

## Author Contributions

AH conceptualized and wrote the manuscript. AA and UV contributed with critical discussion, literature search and improving the final version of the manuscript. All authors contributed to the article and approved the submitted version.

## Conflict of Interest

The authors declare that the research was conducted in the absence of any commercial or financial relationships that could be construed as a potential conflict of interest. The handling editor declared a past collaboration with one of the authors UV.

## References

[1] Freudenberger HJ Staff burn-out. J Soc Issues. (1974) 30:159–65.

[2] Schaufeli WB Buunk BP Burn-out: an overview of 25 years of research theorizing. In: Marc Schabracq J, Jacques Winnubst AM, Cooper CL, editrs. (Hrsg.): The Handbook of Work and Health Psychology. 2. Auflage. John Wiley & Sons: Chichester (2003). p. 382–425. 10.1002/0470013400.ch19

[3] Hillert A Marwitz M Die Burn-out-Epidemie. Oder: Brennt Die Leistungsgesellschaft aus? München: Beck (2006).

[4] Burisch M Das Burn-Out-Syndrom. Theorie der Inneren Erschöpfung (5. Aufl.). Heidelberg: Springer (2014). p. 14–78. 10.1007/978-3-642-36255-2

[5] Maslach C Jackson SE Leiter MP Maslach Burn-Out Inventory Manual, 3rd Edn Palo Alto: Consulting Psychologists Press (1996).

[6] Albrecht A Hillert A Albrecht E Burnout: Coaching versus Psychotherapie PiD. Psychother Dialog. (2018) 19:80–4. 10.1055/a-0556-2563

[7] Spitzer RL Endicott J Robins E. Clinical criteria for psychiatric diagnosis DSM-III. Am J Psychiatry. (1975) 132:1187–92. 10.1176/ajp.132.11.1187 1172654

[8] Spitzer RL. Values and assumptions in the development of DSM-III and DSM-III-R: an insider's perspective and a belated response to sadler, Hulgus, and Agich's “On values in recent American psychiatric classification. J Nervous Ment Dis. (2001) 189:351–9. 10.1097/00005053-200106000-00002 11434635

[9] Burisch M (2001). Available online at: https://www.socialnet.de/lexikon/Burnout-Syndrom (accessed October 14, 2020).

[10] Albrecht E. Business Coaching. Boston: Walter de Gruyter. (2018). p. 26–37. 10.1515/9783110353389

[11] Siegrist J. The effort-reward imbalance model. Occupat Med State Art Rev. (2000) 15:83–87.18444022

[12] Schmitz E Leidl J Brennt wirklich aus, wer entflammt war? Eine LISREL-analyse zum burn-out-prozess bei sozialberufen. Psychol Erziehung Unterricht. (1999) 45:129–42.

[13] Wright J. Stress in the workplace: a coaching approach. Work. (2007) 28:279–84. 17429153

[14] Aronsson G Theorell T Grape T Hammarström A Hogstedt C Marteinsdottir I. A systematic review including meta-analysis of work environment and burn-out symptoms. BMC Public Health. (2017) 17:264. 10.1186/s12889-017-4153-7 28302088 PMC5356239

[15] Shoji K Cieslak R Smuktunowicz E Rogala A Benight C Luszczynska A. Associations between job burn-out and self-efficacy: a meta-analysis. Anxiety Stress Coping. (2016) 29:367–86. 10.1080/10615806.2015.1058369 26080024

[16] Korczak D Kister C Huber B Differentialdiagnostik des Burn-out-Syndroms. Schriftenreihe Health Technology Assessment (HTA) in der Bundesrepublik Deutschland (Bd. 105). Köln: Deutsches Institut für Medizinische Dokumentation und Information (DIMDI) (2010).

[17] Koch S Lehr D Hillert A Burn-out und Chronischer Beruflicher Stress. Göttingen: Hogrefe (2015). 10.1026/02650-000

[18] Leiter MP Durup J The discriminant validity of burn-out and depression. Anxiety Stress Coping. (1994) 7:357–73. 10.1080/10615809408249357

[19] Wurm W Vogel K Holl A Ebner C Bayer D Morkl S. Depression-burn-out overlap in physicians. PLoS ONE. (2016) 11:e0149913. 10.1371/journal.pone.0149913 26930395 PMC4773131

[20] Bianchi R Schonfeld IS Laurent E. Burn-out symptoms: depressive manifestations under psychosocial labels? Asia Pac Psychiatry. (2017) 9:187–202. 10.1111/appy.12280 28856842

[21] Melnick ER Powsner SM Shanafelt TD. In reply—defining physician burn-out, and differentiating between burn-out and depression. Mayo Clin Proc. (2017) 92:1456–8. 10.1016/j.mayocp.2017.07.005 28870365

[22] Schulz P Burn-Out Oder Depression? Wie Sich Entstehung, Prävention und Therapie Beider Störungen Unterscheidet. Lengerich: Pabst (2017).

[23] Deutsche Gesellschaft für Psychiatrie, Psychotherapie, und Nervenheilkunde (Hrsg,.): Positionspapier der Deutschen Gesellschaft für Psychiatrie, Psychotherapie und Nervenheilkunde (DGPPN) zum Thema Burn-out Berlin. (2012). Available online at: https://www.dgppn.de/_Resources/Persistent/7e810b2fd033c8a7d0b13479dc516ad310e11fa1/2012-09-12-dgppn-positionspapier-migration.pdf

[24] Lohmann-Haislah A Stressreport Deutschland. Psychische Anforderungen, Ressourcen und Befinden. Dresden: Bundesanstalt für Arbeitsschutz und Arbeitsmedizin, Dortmund (2012).

[25] Mariotti A. The effects of chronic stress on health: new insights into the molecular mechanisms of brain-body communication. Future Sci OA. (2015) 1:FSO23. 10.4155/fso.15.21 28031896 PMC5137920

[26] Rohland BM Kruse GR Rohrer JE Validation of a single-item measure of burn-out against the maslach burn-out inventory among physicians. Stress Health. (2004) 20:75–9. 10.1002/smi.1002

[27] Hillert A Marwitz M Die Burn-Out-Epidemie. Oder: Brennt Die Leistungsgesellschaft Aus? München: Beck (2006). p. 100–123.

[28] Pines AM Aronson E Kafry D Burn-Out: From Tedium to Personal Grow. New York, NY: Free Press (1981).

[29] Kristensen TS Borritza M Villadsen E Christensen KB The copenhagen burn-out inventory: a new tool for the assessment of burn-out. Work Stress. (2005) 19:192–207. 10.1080/02678370500297720

[30] Milfont TL Denny S Ameratunga S Robinson E Merry S Burn-out and wellbeing: testing the copenhagen burn-out inventory in New Zealand teachers. Soc Indicat Res. (2007) 89:169–77. 10.1007/s11205-007-9229-9

[31] Hillert A Marwitz M Die Burn-Out-Epidemie. Oder: Brennt Die Leistungsgesellschaft Aus? München: Beck (2006).

[32] Maslach C Leiter MP. Understanding the burn-out experience: recent research and its implications for psychiatry. World Psychiatry. (2016) 15:103–11. 10.1111/j.1540-4560.1974.tb00706.x 27265691 PMC4911781

[33] Hillert A Burnout: Zeitbombe oder Luftnummer? Persönliche Strategien und Betriebliches Gesundheitsmanagement Angesichts Globaler Beschleunigung. Stuttgart: Schattauer (2014).

[34] Hakanen JJ Bakker AB Schaufeli WB Burn-out and work engagement among teachers. J Sch Psychol. (2006) 43:495e513 10.1016/j.jsp.2005.11.001

[35] Skaalvik E Skaalvik S Teacher self-efficacy and teacher burn-out: a study of relations. Teach Teacher Educ. (2010) 26:1059–69. 10.1016/j.tate.2009.11.001

[36] Kutsal D Bilge F A study on the burn-out and social support levels of high school students. Educ Sci. (2012) 37:283–97.

[37] Kadi A Ferda Beytekin O Arslan H A research on the burn-out and the teaching profession attitudes of teacher candidates. J Educ Train Stud. (2015) 3:2:107–13. 10.11114/jets.v3i2.677

[38] Gluschkoff K Elovainio M Kinnunen U Mullola S Hintsanen M Keltikangas-Järvinen L. Work stress, poor recovery and burn-out in teachers. Occup Med. (2016) 66:564–70. 10.1093/occmed/kqw086 27412428

[39] Lauermann F König J Teachers professional competence and wellbeing: understanding the links between general pedagogical knowledge, self-efficacy and burn-out. Learn Instr. (2016) 45:9–19. 10.1016/j.learninstruc.2016.06.006

[40] Arvidsson I Leo U Larsson A Håkansson C Persson R Björk J. Burn-out among school teachers: quantitative and qualitative results from a follow-up study in southern Sweden. BMC Public Health. (2019) 19:655. 10.1186/s12889-019-6972-1 31142318 PMC6542045

[41] Rotenstein LS Torre M Ramos MA Rosales RC Guille C Sen S. Prevalence of burn-out among physicians: a systematic review. JAMA. (2018) 320:1131–50. 10.1001/jama.2018.12777 30326495 PMC6233645

[42] Dyrbye LN Burke SE Hardeman RR Herrin J Wittlin NM Yeazel M. Association of clinical specialty with symptoms of burn-out and career choice regret among US resident physicians. JAMA. (2018) 320:1114–30. 10.1001/jama.2018.12615 30422299 PMC6233627

[43] Bellingrath S Kudielka BM Psychobiological pathways from work stress to reduced health: naturalistic experimental studies on the model of effort-reward-imbalance. In: Siegrist J, Wahrendorf M, editors. Work Stress and Health in a Globalized Economy - The Model of Effort-Reward Imbalance. Berlin: Springer (2016). p. 145–70.

[44] Rothe N Steffen J Penza M Kirschbaum C Walther A. Examination of peripheral basal and reactive cortisol levels in major depressive disorder and the burnout syndrome: a systematic review. Neurosci Biobehav Rev. (2020) 14:232–70. 10.1016/j.neubiorev.2020.02.024 32088345

[45] Sennett R Der Flexible Mensch. Kultur des Neuen Kapitalismus (= The Corrosion of Character. New York: W.W: Norton 1998). Berlin: Berlin Verlag (1998).

[46] Rosa H Beschleunigung. Die Veränderung der Zeitstruktur in der Moderne. Frankfurt am Main: Suhrkamp (2012).

[47] Hillert A Gebrauchsanweisung für das Leben in der Postmoderne. Stuttgart: Schattauer/Klett-Cotta (2019).

[48] Bauman Z Liquid Life. Cambridge: Polity Press (2005).

[49] Ehrenberg A Das erschöpfte Selbst. Depression und Gesellschaft in der Gegenwart. Frankfurt am Main: Suhrkamp (La Fatigue d'être soi – dépression et société, 1998). Paris: Odile Jacob (2008).

[50] Rosa H Beschleunigung. Die Veränderung der Zeitstruktur in der Moderne. Frankfurt Main: Suhrkamp (2012).

[51] Freudenberger HJ Richelson G Burnout: The High Cost of High Achievement. New York, NY: Anchor Press (1980).

[52] Bellingrath S Kudielka BM Psychobiological pathways from work stress to reduced health: naturalistic experimental studies on the model of effort-reward-imbalance. In: Siegrist J, Wahrendorf M, editors. Work Stress and Health in a Globalized Economy - The Model of Effort-Reward Imbalance. Berlin: Springer (2016). p. 145–70.

[53] Verhaeghe J Van Den Eede F van den Ameele H Sabbe B Neuro-endocrine correlates of burnout. Tijdschr Psychiatr. (2012) 54:517–26. 22753184

[54] ICD-11 for Mortality Morbidity Statistics Version 04/2019: Available online at: https://www.who.int/mental_health/evidence/burnout

[55] Schmitz E Leidl J Brennt wirklich aus, wer entflammt war? Eine LISREL-analyse zum burn-out-prozess bei sozialberufen. Psychol Erziehung Unterricht. (1999) 45:129–42.

[56] Lohmann-Haislah A Stressreport Deutschland. Psychische Anforderungen, Ressourcen und Befinden. Bundesanstalt für Arbeitsschutz und Arbeitsmedizin. Dresden: Dortmund (2012).

[57] Hillert A Bäcker K Burn-out - a fashionable term or an illness concept? Background and data from the “Stress Monitor” project. JATROS Neurol Psychiatr. (2015) 2:24–7.

[58] Antony MM Bieling PJ Cox BJ Enns MW Swinson RP Psychometric properties of the 42-item and 21-item versions of the depression anxiety stress scales (DASS) in clinical groups and a community sample. Psychol Assess. (1998) 10:176–81. 10.1037/1040-3590.10.2.176

[59] Siegrist J Wege N Pühlhofer F Wahrendorf M. A short generic measure of work stress in the era of globalization: effort-reward imbalance. Int Arch Occup Environ Health. (2008) 82:1005–13. 10.1007/s00420-008-0384-3 19018554

[60] Bianchi R Laurent E Schonfeld IS Bietti LM Mayor E. Memory bias toward emotional information in burnout and depression. J Health Psychol. (2020) 25:1567–75. 10.1177/1359105318765621 29600730

[61] Kiel E Braun A Hillert A Bäcker K Weiß S Gratifikation und befindlichkeit – ein berufsgruppenvergleich von verbeamteten lehrkräften, angestellten im öffentlichen dienst und erwerbstätigen in wirtschaftsunternehmen. Z Arb Wiss. (2019) 73:324–36. 10.1007/s41449-019-00159-w

[62] Hörmann G Laienkonzepte von Gesundheit und Krankheit (Lay concepts of health and disease). In: Laaser U, Sassen G, Murza G, Sabo P, editors. Prävention und Gesundheitsförderung. Heidelberg: Springer (1987). p. 21–33. 10.1007/978-3-642-73096-2_3

[63] Rüdiger J Eirmbter WH Hahn A Laienvorstellungen von Krankheit und Therapie. Ergebnisse einer bundesweiten Repräsentativbefragung (Lay ideas of illness and therapy. Results of a nationwide representative survey). Zeitschr Gesundheitspsychol. (1999) 7:105–19. 10.1026//0943-8149.7.3.105

[64] Hadler NM The illness of work incapacity. Occup Med. (2016) 66:346–8. 10.1093/occmed/kqw060 PMC4913377 27317332

[65] Hofmann B Do health professionals have a prototype concept of disease? The answer is no. Philos Ethics Humanit Med. (2017) 12:6 10.1186/s13010-017-0047-7 28889801 PMC5592715

[66] Seidlein AH Salloch S. Illness and disease: an empirical-ethical viewpoint. BMC Med Ethics. (2019) 20:5. 10.1186/s12910-018-0341-y 30626443 PMC6327539

[67] Vogt H Hofmann B Getz L. Personalized medicine: evidence of normativity in its quantitative definition of health. Theor Med Bioeth. (2016) 37:401–16. 10.1007/s11017-016-9379-3 27638683 PMC5035650

[68] Dyrbye LN Thomas MR Massie FS Power DV Eacker A Harper W. Burn-out and suicidal ideation among U.S. medical students. Ann Intern Med. (2008) 149:334–41. 10.7326/0003-4819-149-5-200809020-00008 18765703

[69] Galán F Ríos-Santos JV Polo J Rios-Carrasco B Bullón P. Burn-out, depression and suicidal ideation in dental students. Med Oral Patol Oral Cir Bucal. (2014) 19:e206–11. 10.4317/medoral.19281 24121916 PMC4048106

[70] Wörfel F Gusy B Lohmann K Kleinber D Validierung der deutschen kurzversion des maslach-burn-out-inventars für studierende. Z Gesundheitspsychol. (2015) 23:191–6. 10.1026/0943-8149/a000146

[71] Hillert S Wörfel F Weiß S Exposure and burn-out experience of pupils in the 5th - 10th grade of a bavarian gymnasium. Influence of framework conditions and individual goals. Präv Rehabil. (2018) 30:83–90. 10.5414/PRX0528

[72] Kutsal D Bilge F A study on the burn-out and social support levels of high school students. Educ Sci. (2012) 37:283–97.

[73] McGonagle AK Beatty JE Joffe R. Coaching for workers with chronic illness: evaluating an intervention. J Occupat Health Psychol. (2014) 19:385–98. 10.1037/a0036601 24796227

[74] Grant AM Solution-focused cognitive-behavioral coaching for sustainable high performance and circumventing stress, fatigue, and burnout. Consulti Psychol J. (2017) 69:98–111. 10.1037/cpb0000086

[75] Hillert A Albrecht A Burn-out – Stress. Depression. Interdisziplinäre Strategien für Ärzte, Therapeuten und Coaches. München: Elsevier (2020).

